# Development and Validation of a Deep Learning Model for Non–Small Cell Lung Cancer Survival

**DOI:** 10.1001/jamanetworkopen.2020.5842

**Published:** 2020-06-03

**Authors:** Yunlang She, Zhuochen Jin, Junqi Wu, Jiajun Deng, Lei Zhang, Hang Su, Gening Jiang, Haipeng Liu, Dong Xie, Nan Cao, Yijiu Ren, Chang Chen

**Affiliations:** 1 Department of Thoracic Surgery, Shanghai Pulmonary Hospital, Tongji University School of Medicine, Shanghai, China; 2 College of Design and Innovation, Tongji University, Shanghai, China; 3 Shanghai Key Laboratory of Tuberculosis, Shanghai Pulmonary Hospital, Tongji University School of Medicine, Shanghai, China; 4 Computer Science, NYU Shanghai, Shanghai, China

## Abstract

**Importance:**

There is a lack of studies exploring the performance of a deep learning survival neural network in non–small cell lung cancer (NSCLC).

**Objectives:**

To compare the performances of DeepSurv, a deep learning survival neural network with a tumor, node, and metastasis staging system in the prediction of survival and test the reliability of individual treatment recommendations provided by the deep learning survival neural network.

**Design, Setting, and Participants:**

In this population-based cohort study, a deep learning–based algorithm was developed and validated using consecutive cases of newly diagnosed stages I to IV NSCLC between January 2010 and December 2015 in a Surveillance, Epidemiology, and End Results database. A total of 127 features, including patient characteristics, tumor stage, and treatment strategies, were assessed for analysis. The algorithm was externally validated on an independent test cohort, comprising 1182 patients with stage I to III NSCLC diagnosed between January 2009 and December 2013 in Shanghai Pulmonary Hospital. Analysis began January 2018 and ended June 2019.

**Main Outcomes and Measures:**

The deep learning survival neural network model was compared with the tumor, node, and metastasis staging system for lung cancer–specific survival. The C statistic was used to assess the performance of models. A user-friendly interface was provided to facilitate the survival predictions and treatment recommendations of the deep learning survival neural network model.

**Results:**

Of 17 322 patients with NSCLC included in the study, 13 361 (77.1%) were white and the median (interquartile range) age was 68 (61-74) years. The majority of tumors were stage I disease (10 273 [59.3%]) and adenocarcinoma (11 985 [69.2%]). The median (interquartile range) follow-up time was 24 (10-43) months. There were 3119 patients who had lung cancer–related death during the follow-up period. The deep learning survival neural network model showed more promising results in the prediction of lung cancer–specific survival than the tumor, node, and metastasis stage on the test data set (C statistic = 0.739 vs 0.706). The population who received the recommended treatments had superior survival rates than those who received treatments not recommended (hazard ratio, 2.99; 95% CI, 2.49-3.59; *P* < .001), which was verified by propensity score–matched groups. The deep learning survival neural network model visualization was realized by a user-friendly graphic interface.

**Conclusions and Relevance:**

The deep learning survival neural network model shows potential benefits in prognostic evaluation and treatment recommendation with respect to lung cancer–specific survival. This novel analytical approach may provide reliable individual survival information and treatment recommendations.

## Key Points

**Question:**

Can deep learning architecture be applied for individual prognosis evaluation and treatment recommendation?

**Findings:**

In this cohort study of 17 322 patients with non–small cell lung cancer. The performance of a deep learning model was assessed on real-life clinical data sets. The ability of a deep learning model to learn complex associations between an individual’s characteristics and the outcome benefits of different treatments was also elucidated; particularly, a deep learning network identified persons with non–small cell lung cancer and survival more accurately than tumor, node, metastasis staging.

**Meaning:**

Findings suggest that this novel analytical approach may have great potential in providing individual prognostic information and treatment recommendations in real clinical scenarios.

## Teaser

This cohort study compares the performances of a deep learning survival neural network with a tumor, node, and metastasis staging system in the prediction of survival.

## Introduction

Lung cancer is the most commonly diagnosed cancer in China and the second in the United States, approximately 85% of which is non–small cell lung cancer (NSCLC).^1^ The precise stratification of patients with NSCLC into groups according to survival outcomes represents a crucial step in treatment. The staging system in the 8th edition of the American Joint Committee on Cancer classifies patients based on tumor, node, and metastasis (TNM) staging.^2^ However, the survival rate within the same stage cohort varies widely.^3,4,5^ It has been found that other independent prognostic factors including age, sex, histology, and treatment choices could significantly contribute to individualized predictions of survival.^6^

To improve the precision of lung cancer survival estimations, Cox proportional hazard models have gained popularity as a way of predicting outcomes.^7^ For example, the nomogram is a reliable tool that has demonstrated the ability to quantify risk by combining and clarifying significant clinical characteristics for clinical oncology.^6^ By drafting a concise chart of an outcome-risk predictive model, a nomogram derives the risk probability of a specific event, such as lung cancer–specific survival (LCSS). In various cancers, nomograms possess the ability to derive more precise risk predictions when incorporated with TNM staging.^8,9^ However, these models have several limitations with respect to time-to-event prediction for the clinical management of patients with cancer, including the precise evaluation of overall survival and time to progression.^10^ Moreover, these models make linearity assumptions rather than perform nonlinear analyses that reflect real-world clinical characteristics.^11^ Therefore, there is a need for better solutions that focus on nonlinear variables.^12^

Deep learning networks can learn the highly intricate and linear/nonlinear associations between prognostic clinical characteristics and an individual’s risk of death from LCSS.^13^ In application, these networks have even shown potential for providing individual recommendations based on the calculated risk.^14^ For example, by analyzing clinical data in the Surveillance, Epidemiology, and End Results (SEER) cancer registry, Bergquist et al^15^ assembled computerized methods including random forests, lasso regression, and neural networks to achieve 93% accuracy in predicting lung cancer stages. In another study, Corey et al^16^ developed a software package (Pythia) based on machine learning models that incorporated a patient’s age, sex, clinical baseline, race/ethnicity, and comorbidity history to determine the risk of postoperative complications or deaths. Matsuo et al^17^ also developed a deep learning network model that has demonstrated a higher C statistic than the traditional proportional hazard regression model (C statistic = 0.795 vs 0.784) for progression-free survival analysis. Furthermore, Katzman et al^18^ developed a novel deep learning method for survival analysis that uses a deep learning network to integrate Cox proportional hazards, which is referred to as the learning survival neural network (DeepSurv). The authors demonstrated that DeepSurv performed as well as published survival models and could be used to provide treatment recommendations for better survival outcomes.

The present study design follows the American Joint Committee on Cancer criteria for model adoption and the transparent report of a deep learning architecture for individual prognosis and treatment recommendation. In this study, we first describe the performance of DeepSurv^18^ on real-life clinical data sets. Second, we elucidate how the DeepSurv model can learn complex associations between an individual’s characteristics and the outcome benefits of different treatments.

## Methods

### Eligibility Criteria and Clinical Information

All patients gave informed oral consent prior to data collection. After obtaining institutional review board approval from Shanghai Pulmonary Hospital, we selected patients from the SEER 18 Regs Research Data + Hurricane Katrina Impacted Louisiana Cases, Nov 2017 Sub, which includes clinical records on cancer occurrences in 18 areas of the United States and contains approximately 27.8% of the population. Clinical cases were included if the following criteria were met: pathologically confirmed primary stage I to IV NSCLC (only adenocarcinoma and squamous cell carcinoma) between January 2010 and December 2015 and the presence of 1 malignant primary lesion. From the SEER database (eTable 1 in the Supplement), we collected the baseline information of cases (sex, age, and marriage status), tumor characteristics (location, size, histologic grade, histologic type, TNM stage, SEER code (CS extension, CS mets at dx, regional nodes examined, regional nodes positive, lung–pleural/elastic layer invasion by H and E or elastic stain, lung–separate tumor nodules–ipsilateral lung, lung–surgery to primary site]), and treatment details (surgical type).^19,20^ Patients were excluded if any of the included clinical characteristics status were unknown or missing. The outcome of interest in this study included LCSS according to specific codes provided by SEER (defined as the interval from surgery until death as a result of lung cancer). These patients were randomly divided into the training and validation cohort at a ratio of 8 to 2. To validate the DeepSurv model, an external test cohort was provided by the CHINA database. The cohort comprised 1182 patients with stage I to III NSCLC diagnosed between January 2009 and December 2013 in Shanghai Pulmonary Hospital, which are completely distinct from the patients in SEER database. This study followed the Strengthening the Reporting of Observational Studies in Epidemiology (STROBE) reporting guideline.

### Deep Learning Model Design

**Figure 1.**
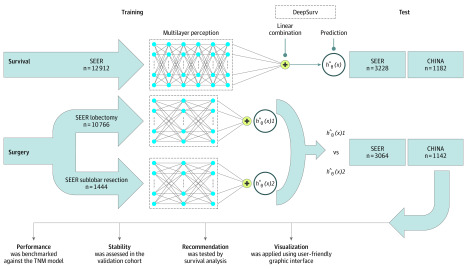
Diagram of the Study Procedure Deep learning networks were trained end to end on 3 data set groups. The training and testing of these networks were all conducted on independent data sets. Four further experiments were conducted on the networks to test their performances against tumor, node, and metastasis (TNM) models, assess their degrees of stability, formulate recommendations, and finally, accomplish model visualization. SEER indicates Surveillance, Epidemiology, and End Results.

In this study, DeepSurv was used to analyze patient-individual survival outcomes, which is a deep learning algorithm that can predict individual survival risk values (Figure 1).^18^ We use deep feed-forward neural network and the Cox proportional hazards model in survival analysis. The DeepSurv model contained a core hierarchical structure with fully connected feed-forward neural networks with a single output node to calculate the survival risks *hθ*(x_i_) of patients using the negative log-partial likelihood function. More details about the DeepSurv were described in the eMethods in the Supplement. Using the provided data set, we compared the performances of the TNM staging model and our deep learning model with respect to 2 tasks (LCSS predictions) with 3 different data sets (Figure 1).

### Data Analysis

First, we developed a 6-layer neural network for predicting patient LCSS in the NSCLC training data set (n = 12 912). To validate the prediction performance, we used Harrell C statistic and calibration plots to evaluate the network discrimination and calibration in the NSCLC validation data set (n = 3228) and CHINA data set (n = 1182).

Next, we trained a personalized treatment recommendation system using separately developed lobar and sublobar resection risk prediction models with a 3-layer neural network in the lobectomy (n = 10 766) and sublobectomy (n = 1444) training data sets. For each patient in the lobectomy and sublobectomy validation data set (SEER: n = 3064; CHINA: n = 1142), we chose the lower-risk value of the model’s treatment as the recommendation.

Finally, we categorized the patients into 2 groups according to the consistency of the treatment received and recommended. For survival analysis, we used the Kaplan-Meier method to analyze LCSS between different groups and the log-rank test to compare survival curves.

An additional Cox proportional hazard regression model with nonneural network methods^6,17^ was performed following the simple backward-stepwise approach using all the variables included in the DeepSurv model. It estimated the risk function h(x_i_) of the event occurring (LCSS) for patient i based on included features x_i_ using a linear function: h(Xi) = ∑ x_i_β_i_ (β = the coefficient of x_i_).

### Model Visualization

We also developed a user-friendly interface to facilitate the survival predictions and treatment recommendations of the DeepSurv model. This interface consists of 3 views: (1) the user input view, (2) the survival prediction view, and (3) the treatment recommendation view. The user input view is designed to help users input all entries regarding patient characteristics using the XML schema constructed based on the features input into DeepSurv models. The user input view allows users to predict the survival probability and obtain a treatment recommendation based on specific patient information by clicking the predict and recommendation buttons, respectively. All SEER codes followed the SEER guideline.^21^

### Statistical Analysis

A 2-sided *P* value less than .05 was considered to be statistically significant. The Akaike information criterion was calculated to assess the risk of overfitting. The likelihood-based method was applied to the type I censoring design.^22^ All statistical analyses were performed with SPSS version 23 (IBM Corporation) software. The C statistic was performed by comparing C package with R statistical software (R Project for Statistical Computing), and the survival curves were plotted using GraphPad Prism 7 (GraphPad Software) software. Codes in our study are available online (https://github.com/thoraciclang/Deep_Lung).^23^ Analysis began January 2018 and ended June 2019.

## Results

### Screening Process and Clinicopathology

A total of 17 322 patients with NSCLC were included in the study. According to the screening criteria, a total of 16 140 patients diagnosed as having NSCLC from the SEER database were included (eFigure 1A in the Supplement). Table 1 shows the patients’ main baseline clinical characteristics (eTables 2 and 3 in the Supplement). The majority of patients were white (13 361 [82.8%]), and the median (interquartile range) age was 68 (61-74) years. The majority of tumors were stage I disease (9327 [57.8%]) and adenocarcinoma (11 037 [68.4%]). The median (interquartile range) follow-up time was 24 (10-43) months. There were 2893 patients (17.9%) who had events (deaths from NSCLC) during the follow-up time. There were 1182 patients diagnosed with NSCLC from CHINA database (eFigure 1B in the Supplement). There were 226 events (deaths from NSCLC) over a median (interquartile range) follow-up time of 63.3 (53-70) months.

**Table 1.** Main Characteristics of Patients in the Whole Data Sets of Survival Analysis

Characteristic^a^	Data set, No. (%)		
	Training	SEER (test 1)	CHINA (test 2)
Age at diagnosis, median (range), y	68 (28-95)	68 (19-92)	60 (30-87)
Sex			
Female	6657 (51.6)	1639 (50.8)	642 (54.3)
Male	6255 (48.4)	1589 (49.2)	540 (45.7)
Histologic type			
Adenocarcinoma	8794 (68.1)	2243 (69.5)	948 (80.2)
Squamous cell carcinoma	4118 (31.9)	985 (30.5)	234 (19.8)
Marital status at diagnosis			
Unmarried	5304 (41.1)	1843 (57.1)	526 (44.5)
Married	7608 (58.9)	1385 (42.9)	656 (55.5)
T			
T1a	563 (4.4)	139 (4.3)	128 (10.8)
T1b	3156 (24.4)	804 (24.9)	396 (33.5)
T1c	2342 (18.1)	641 (19.9)	346 (29.3)
T2a	3258 (25.2)	791 (24.5)	208 (17.6)
T2b	594 (4.6)	141 (4.4)	56 (4.7)
T3	1994 (15.4)	445 (13.8)	40 (3.4)
T4	1005 (7.8)	267 (8.3)	8 (0.7)
N			
N0	9712 (75.2)	2439 (75.6)	1030 (87.1)
N1	1732 (13.4)	418 (12.9)	54 (4.6)
N2	1422 (11)	356 (11)	98 (8.3)
N3	46 (0.4)	15 (0.5)	0
M			
M0	12 559 (97.3)	3132 (97)	1182 (100)
M1a	143 (1.1)	41 (1.3)	0
M1b	202 (1.6)	52 (1.6)	0
M1c	8 (0.1)	3 (0.1)	0
LCCS			
Alive	10 581 (81.9)	2666 (82.6)	956 (80.9)
Dead	2331 (18.1)	562 (17.4)	226 (19.1)
Surgery to primary site			
Pneumonectomy	613 (4.7)	132 (4.1)	40 (3.4)
Lobectomy	10 766 (83.4)	2695 (83.5)	872 (73.8)
Sublobar	1444 (11.2)	369 (11.4)	270 (22.8)
None	89 (0.7)	32 (1.0)	0

Abbreviations: LCCS, lung cancer–specific survival; M, metastasis; N, node; SEER, Surveillance, Epidemiology, and End Results cancer registry; T, tumor.

^a^ Other detailed clinical characteristics can be found in eTables 2 and 3 in the Supplement.

### Training Curves

eFigure 2 in the Supplement demonstrated the training curves of networks in 3 submodels. The accuracy during the training course was indicated by validation and training lines. The curve was plotted to monitor the training course as the weights of the network were adjusted over each epoch, which represented the algorithm runs through the entire training and test data sets. After fine tuning, the change trend of loss and accuracy tended to become smoother and the algorithm maintained high accuracy on the validation set without significant overfitting. With a 500-epoch limit, we chose the model with the best performance on the test data set.

### Calibration and Validation of the Prognostic DeepSurv for LCSS in the Test Set

**Figure 2.**
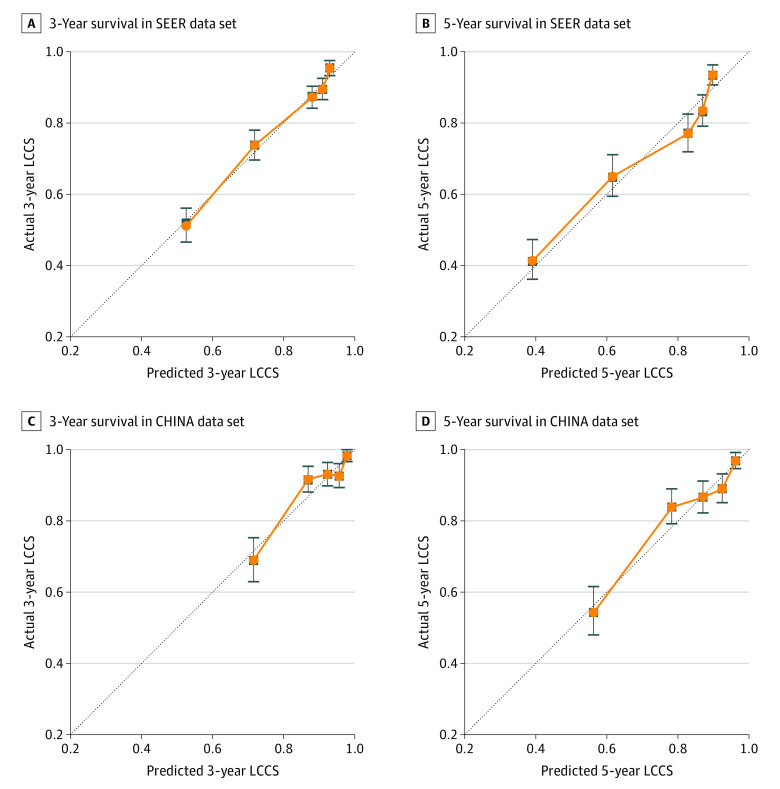
Calibration Plots for Lung Cancer–Specific Survival (LCCS) for the DeepSurv Model Three-year survival (A) and 5-year survival (B) of Surveillance, Epidemiology, and End Results (SEER) data set and 3-year survival (C) and 5-year survival (D) of CHINA data set are shown.

We compared the TNM staging model to DeepSurv for LCSS in the test data sets (Table 2). The calibration plot indicated the calibration and how far the predictions of DeepSurv deviated from the actual event (Figure 2). In general, the actual outcomes in our databases of all patients with NSCLC for 3-year and 5-year LCSS were highly consistent with those predicted by the DeepSurv model, with most points falling almost directly on the 45° line. The DeepSurv model generated significantly better predictions than the TNM staging model (C statistic for TNM stage vs DeepSurv = 0.70; 95% CI, 0.681-0.731 vs 0.739; 95% CI, 0.713-0.764 [*P* < .001]). In the test group (CHINA data set), the DeepSurv model (C statistic = 0.742; 95% CI, 0.709-0.775) showed significantly better prediction than TNM model (C statistic = 0.706; 95% CI, 0.681-0.731; *P* < .001). High C statistic was observed for the results of the lobectomy and sublobar resection test data sets (Table 2). The feature component weightings in DeepSurv model are listed at eTable 4 in the Supplement.

**Table 2.** Comparison of TNM Stage and DeepSurv Model for Survival Prediction in Test Data Sets

LCCS outcome	Model	C statistic (95% CI)	*P* value
SEER	TNM	0.706 (0.681-0.731)	NA
	DeepSurv	0.739 (0.713-0.764)	<.001
CHINA	TNM	0.691 (0.659-0.724)	NA
	DeepSurv	0.742 (0.709-0.775)	<.001
Treatment	DeepSurv (lobectomy, SEER)	0.725 (0.698-0.751)	NA
	DeepSurv (sublobar resection, SEER)	0.698 (0.672-0.725)	NA

Abbreviations: LCCS, lung cancer-specific survival; NA, not applicable; SEER, Surveillance, Epidemiology, and End Results cancer registry; TNM, tumor, node, and metastasis.

The Cox proportional hazard regression model (eTable 5 in the Supplement) was compared with the DeepSurv model for LCSS. The DeepSurv model had significantly better predictions compared with the Cox proportional hazard regression model (C statistic for Cox proportional hazard regression model vs DeepSurv model = 0.716; 95% CI, 0.705-0.727 vs 0.739; 95% CI, 0.713-0.764). The Akaike information criterion value of TNM stage model, Cox proportional hazard regression model, and DeepSurv model were 10741.89, 10307.08, and 10310.52, respectively.

### Treatment Recommender

First, we plotted 2 Kaplan-Meier survival curves: the outcome of test cases whose actual treatments were the same as those recommended and those whose were not (eFigure 3 in the Supplement). The population that followed the recommendation experienced significantly better survival rates than those who did not (SEER: hazard ratio [HR], 2.99; 95% CI, 2.49-3.59; *P* < .001 vs CHINA: HR, 2.14; 95% CI, 1.65-2.77; *P* < .001). Furthermore, patients in the test data sets were classified into lobectomy and sublobar resection groups according to the received treatment. Consistent with prior analyses, LCSS favored lobectomy compared with sublobar resection in the subgroup with the lobectomy recommendation (SEER: HR, 1.79; 95% CI, 1.28-2.50; *P* = .001 vs CHINA: HR, 1.92; 95% CI, 1.30-2.83; *P* = .001). No significant distinction in survival results were observed for lobectomy and sublobar resection in the subgroup with the sublobar resection recommendation (SEER: HR, 0.65; 95% CI, 0.41-1.02; *P* = .06 vs CHINA: HR, 0.75; 95% CI, 0.44-1.77; *P* = .28).

### Model Visualization

**Figure 3.**
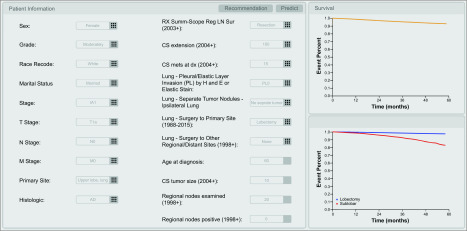
User-Friendly Interface of DeepSurv Model, Which Facilitates Survival Prediction and Treatment Recommendation

In the prediction view, the system invokes a prediction model (Figure 3; Video), and the DeepSurv model is used to predict patients’ survival probability. The analysis results are visualized in a graphic view as a survival curve, which indicates the survival probability of the patient input over time. In the recommendation view, a recommendation model is adopted by the system, which can provide different patient survival probabilities for different treatments (lobectomy or sublobar resection) (Figure 3; Video). The survival curves of lobectomy and sublobar resection are also presented in a graphic view to facilitate visual comparison.

**Video.** Deep Learning–Based Lung Cancer Survival Prediction Model User interface for a deep learning–based algorithm that predicts lung cancer–specific survival based on patients’ demographic characteristics, tumor stage and lymph node status, and prior treatment.

## Discussion

The results of our pilot study proved that the deep learning network model (DeepSurv) performed better than conventional linear regression modeling (TNM staging model) in postoperative outcome prediction for patients with newly diagnosed NSCLC. Also, this model may serve as a useful analytical tool for treatment recommendation in patients with NSCLC, given its evidence of the significant prognostic benefits of following the treatment recommendation, which clearly outweigh those associated with not following the recommendation.

Previous studies have reported a series of linear models to predict the survival of patients with lung cancer.^24,25,26,27^ However, few risk factors have been selected to these models, which is significantly associated with the survival or recurrence. For example, Liang et al^25^ constructed a nomogram based on 6 factors. On the other hand, our Cox analysis demonstrated the contribution of 16 factors in the DeepSurv model. Obviously, a more comprehensive analysis could be performed by a nonlinear deep learning model. After reviewing the most relevant advanced research, we found many studies to have already applied deep learning models in their analytical approaches to surgical oncology research.^13^ However, most studies have focused on diagnostic application,^28,29,30^ such as radiographic image automated quantification,^14,31,32,33,34,35^ digital histopathology image interpretation,^30,36,37,38,39^ or biomarker analysis.^11,40^ Examples of published research using deep learning models for prognostic prediction in surgical oncology are rare, to our knowledge. In NSCLC research, the deep learning technique has been applied to digital histopathology image interpretation, driver mutation risk detection,^40^ and image characteristics discrimination,^33,41^ but only a few studies have focused on postoperative outcome prediction or surgical recommendation, to our knowledge.

As a new analytic tool, the deep learning network model will likely become more widely applied to support clinical decision-making.^13^ The performance of deep learning models in improving treatment outcomes is a key question and requires solid validation in the real world. In an analysis of 1194 patients with NSCLC, Hosny et al^14^ evaluated the prognostic signatures of quantitative imaging features, which were extracted using deep learning networks. Based on their study of the TNM stages of postoperative patients, the authors’ main finding was that deep learning networks significantly outperformed previous models. In our study, we selected a larger patient cohort with NSCLC of unselected consecutive cases including I to IV stages for model training and testing, which provide more solid results for interpretation. The advantages of the deep learning network model for postoperative outcome prediction in surgical research can be summarized as follows.^42,43^ First, DeepSurv shows improved adaptability to variables with a nonlinear association, which includes real-world clinical factors. Unlike other models, deep learning algorithms can integrate the nonlinear risk functions associated with outcomes. Second, DeepSurv possesses flexibility in dealing with complex clinical factors. DeepSurv models cannot only automatically learn feature representations from uninterpreted clinical data but also analyze censored factors. Also, the predictions of the DeepSurv model have been proven to perform better in big data analysis. Owing to its ability to learn factor representation, the advantages of the DeepSurv model in dealing with both large factors and sample size may play a big role in biomedical marker analyses.^44,45,46^

It is a surgeon’s duty to introduce clinical information to patients. To facilitate discussion of different potential surgical options, surgeons and patients need an informative tool that focuses on survival benefits. In real cases, the establishment of a user-friendly graphic interface based on a patient communication framework will be key to effectively conveying results and illustrating complex analyses, including prognostic prediction, treatment recommendation to patients and family members, and improving the surgeons’ understanding of deep learning models.^44,47^ With its fast application and convenient operation, the user-friendly graphic interface example established in our study (Figure 3; Video) shows potential for use with any type of surgical care. To date, identifying patients who are appropriate for initial surgical management and conveying individualized prognostic analyses of postoperative outcomes has been an elusive goal. Instead, most published models are guided by patient characteristics to generate prognostic factors and are influenced by biases for different treatments.^48^ The DeepSurv model and its user-friendly graphic interface has the potential to address this clinical dilemma and better share individual outcomes following different surgical procedures.

### Limitations

Since the innovation of deep learning models, many limitations have been recognized. First, deep learning network models are computationally expensive to train and validate. The process of predictions can be hard to interpret because the deep learning networks function much like black boxes, which make it difficult to determine how the network arrives at its decisions. We also recognize that single-clinical data sources have limited clinical characteristics compared with the automated quantification of radiographic images. In this study, we examined 127 features of 21 characteristics in the model. Some important factors including preoperative elements were neglected, which makes the recommendation system need more improvements and stay at feasibility trial status. Also, external validation is lacking in this study. Further study is needed to validate the advantages of deep learning networks in survival prediction.

## Conclusions

To our knowledge, this study is the first to explore the performance of a deep learning network that integrates Cox proportional hazards (DeepSurv) in NSCLC and to obtain promising results in outcome prediction. In addition, we demonstrated DeepSurv’s potential to provide personalized treatment recommendations based on real clinical data.

## Notes

Supplement. **eMethods.** **eTable 1.** All Clinical Features Integrated in the Model **eTable 2.** Characteristics of Patients in the Training Dataset of Survival Analysis **eTable 3.** Characteristics of Patients in the Test Dataset of Survival Analysis **eTable 4.** Feature Component Weightings in the DeepSurv Model **eTable 5.** Survival Predictors in Cox PH Model **eFigure 1.** Flow chart of datasets construction. (A) SEER dataset, (B) CHINA dataset **eFigure 2.** Training curves of networks in the survival dataset of SEER database (A), lobectomy dataset (B), and sublobar resection dataset (C) **eFigure 3.** Lung Cancer–Specific Survival Recommendation Comparisons of SEER Data set (A), SEER Lobectomy Test Data set (B), and SEER Sublobar Resection Test Data set (C); Lung Cancer–Specific Survival Recommendation Comparisons of CHINA Data set (D), CHINA Lobectomy Test Data set (E), and CHINA sublobar Resection Test Data set (F)
