# Supplementary material for: Development and Validation of a Deep Learning Model for Non–Small Cell Lung Cancer Survival
Source: JAMA Netw Open. 2020 Jun 3;3(6):e205842. doi: 10.1001/jamanetworkopen.2020.5842 (PMC7272121; doi:10.1001/jamanetworkopen.2020.5842)
Supplement: Supplement. — eMethods. eTable 1. All Clinical Features Integrated in the Model eTable 2. Characteristics of Patients in the Training Dataset of Survival Analysis eTable 3. Characteristics of Patients in the Test Dataset of Survival Analysis eTable 4. Feature Component Weightings in the DeepSurv Model eTable 5. Survival Predictors in Cox PH Model eFigure 1. Flow chart of datasets construction. (A) SEER dataset, (B) CHINA dataset eFigure 2. Training curves of networks in the survival dataset of SEER database (A), lobectomy dataset (B), and sublobar resection dataset (C) eFigure 3. Lung Cancer–Specific Survival Recommendation Comparisons of SEER Data set (A), SEER Lobectomy Test Data set (B), and SEER Sublobar Resection Test Data set (C); Lung Cancer–Specific Survival Recommendation Comparisons of CHINA Data set (D), CHINA Lobectomy Test Data set (E), and CHINA sublobar Resection Test Data set (F) [file jamanetwopen-3-e205842-s001.pdf]

Supplementary Online Content

She Y, Jin Z, Wu J, et al. Development and validation of a deep learning model for non–small cell lung cancer survival. *JAMA Netw Open*. 2020;3(6):e205842. doi:10.1001/jamanetworkopen.2020.5842

eMethods.
eTable 1. All Clinical Features Integrated in the Model
eTable 2. Characteristics of Patients in the Training Dataset of Survival Analysis
eTable 3. Characteristics of Patients in the Test Dataset of Survival Analysis
eTable 4. Feature Component Weightings in the DeepSurv Model
eTable 5. Survival Predictors in Cox PH Model
eFigure 1. Flow chart of datasets construction. (A) SEER dataset, (B) CHINA dataset
eFigure 2. Training curves of networks in the survival dataset of SEER database (A), lobectomy dataset (B), and sublobar resection dataset (C)
eFigure 3. Lung Cancer–Specific Survival Recommendation Comparisons of SEER Data set (A), SEER Lobectomy Test Data set (B), and SEER Sublobar Resection Test Data set (C); Lung Cancer–Specific Survival Recommendation Comparisons of CHINA Data set (D), CHINA Lobectomy Test Data set (E), and CHINA sublobar Resection Test Data set (F)

This supplementary material has been provided by the authors to give readers additional information about their work.

© 2020 She et al. *JAMA Network Open.*

eMethods.

1 Survival data

Survival data consists of three parts: patient information X, a failure event time T, and an event label E. If a failure is observed, the failure time T is the duration between the time in which the patient information was collected and the time of the failure occurring, the event label equals to 1. If no failure is observed, the failure time T is the duration between the information collected time and the last contact time with the patient, the event label is 0.

2 Survival Analysis Model

We use deep feed-forward neural network and the Cox proportional hazards model in survival analysis.

2.1 The Cox Proportional Hazards Model

The Cox proportional hazards model is a typical method for individual's survival analysis based on hazards function. Specially, the hazard function $\lambda(t)$ is the probability that an individual will survival up to time t and pass out between time t and time $t + \delta$.

$$\lambda(t) = \lim_{\delta \to 0} \frac{Pr(t \leq T < t + \delta | T \geq t)}{\delta}$$

The Cox model assumes that the hazards function can be modeled as a baseline hazards function $\lambda_0(t)$ and the risk level $l_x = e^{h(x)}$. $h(x)$ is denoted as the log-risk function. The form of the hazards function is as follow:

$$\lambda(t|x) = \lambda_0(t) \cdot e^{h(x)}$$

2.2 The Deep Neural Network Structure

We further apply deep learning technique in Cox proportional hazards analysis. A deep neural network is trained to model the log-risk function $h(x)$ in the hazards function. The network takes patient's information x as input. Several

© 2020 She et al. *JAMA Network Open.*

fully-connected layers followed by the dropout layers embed the input into a hidden vector. After that, the hidden vector is fed into the output layer which contains a linear activation function to estimate the log-risk function.

The loss function of the model is set to be the Cox partial likelihood with regularization:

$$l(\theta) := -\frac{1}{N_{E=1}} \sum_{i:E_i=1} \left(h_\theta(x_i) - \log \sum_{j \in \Re(T_i)} e^{h_\theta(x_j)} \right) + \alpha \cdot \|\theta\|_2^2$$

where θ is the weights in neural network, α is the regularization parameter, $h_\theta(x)$ is the output of the network, $N_{E=1}$ is the number of patients with observable failures, and $\Re(t) = \{i : T_i > t\}$ is the set of patient who are at risk of failure at time t. Adaptive Moment Estimation (Adam) for the gradient descent algorithm is applied to optimize the loss function.

2.3 The Survival Probability Curve

According to assumption of the Cox model, a baseline hazards function needs to be estimated for modeling individual's survival probability curve as follow:

$$\lambda_0(t) = 1 - \frac{\sum_{i \in \Im(t)} 1}{\sum_{i \in \Im(t)} 1 + \sum_{i \in \Re(t)} e^{h_\theta(x_i)}}$$

where $\Im(t) = \{i : T_i <= t; E_i = 1\}$

3 Treatment Recommendation

In clinical scenario, the treatment patients undergo has an important impact on patients' survival probability. In general, we consider that each treatment k has its own risk function $e^{h_k(x)}$. Thus, the hazard function of treatment k is:

$$\lambda(t; x | Treatment = k) = \lambda_0(t) \cdot e^{h_t(x)}$$

Considering two treatments k_i and k_j, we can compare the survival curves of treatment k_i and k_i to explore the probability difference between these two treatment at multiple time points.

© 2020 She et al. *JAMA Network Open.*

eTable 1. All Clinical Features Integrated in the Model

Characteristics	Features										
Sex	Female	Male									
Primary Site	Main bronchus/Carina/Hilum/Bronchus intermedius	Upper lobe, lung	Middle lobe, lung	Lower lobe, lung	Overlapping lesion of lung	Lung, NOS/Bronchus, NOS					
Histologic Type	AD	SD									
Grade	Moderately	Poor	undifine	well							
Stage	IA1	IA2	IA3	IB	IIA	IIB	IIIA	IIIB	IIIC	IVA	IVB
T stage	T1a	T1b	T1c	T2a	T2b	T3	T4				
N stage	N0	N1	N2	N3							
M stage	M0	M1a	M1b	M1c							
RX Summ--Scope Reg LN Sur (2003+)	None	Biopsy	Resection	System resection							
CS tumor size (2004+)	1-988mm	Microscopic focus or foci only and no size of focus given	Diffuse (entire lobe)								
CS extension (2004+)	In situ, intraepithelial, noninvasive	Tumor confined to one lung	Superficial tumor of any size with invasive component limited to bronchial wall, with or without proximal extension to the main stem bronchus	Stated as T1a with no other information on extension	Stated as T1b with no other information on extension	Stated as T1[NOS] with no other information on extension	Extension from other parts of lung to main stem bronchus, NOS	Tumor involving main stem bronchus, NOS	Direct tumor invasion into an adjacent ipsilateral lobe	Tumor confined to hilus	Tumor confined to carina
CS mets at dx (2004+)	No distant metastasis	Distant lymph node(s), including cervical nodes	Malignant pleural effusion, ipsilateral or same lung	Malignant pleural effusion, contralateral or other lung	Malignant pleural effusion, ipsilateral and contralateral lungs (Bilateral pleural effusion)	Malignant pleural effusion, unknown if ipsilateral or contralateral lung	Malignant pericardial effusion	Malignant pericardial effusion plus contralateral or bilateral pleural effusion	Extension to:Contralateral lung/Contralateral main stem bronchus/Separate tumor nodule(s) in contralateral lung/Pleural tumor foci or nodules on contralateral lung	Pleural tumor foci or nodules on the ipsilateral lung separate from direct invasion	Extension to contralateral lung plus pleural or pericardial effusion or separate pleural tumor foci
Regional nodes examined (1988+)	1-100										
Regional nodes positive (1988+)	1-100										
Race recode	White	African Americans	American Indian/Alaska Native	Asian or Pacific Islander							
Age at diagnosis	1-120										
Marital status at diagnosis married=1 un-married=0	married	un-married									
Lung - Pleural/Elastic Layer Invasion (PL) by H and E or Elastic Stain	PL0	PL1	PL2	PL3							
Lung - Separate Tumor Nodules - Ipsilateral Lung	No separate tumor nodules noted	Separate tumor nodules in ipsilateral lung, different lobe	Separate tumor nodules in ipsilateral lung, same lobe	Separate tumor nodules, ipsilateral lung, same and different lobe							
Lung - Surgery to Primary Site (1988-2015)	Peumonectomy	Lobectomy	Sublobar	None							
Lung - Surgery to Other Regional/Distant Sites (1998+)	None	Surgery To Distant Site Or Nodes	Surgery To Regional Site								

Characteristics																																								
CS extension (2004+)	Localized, NOS	Atelectasis/obstructive pneumonitis that extends to the hilar region but does not involve the entire lung Or atelectasis/obstructive pneumonitis, NOS	Extension to but not into pleura, including invasion of elastic layer/BUT not through the elastic layer	Invasion of pleura, including invasion through the elastic layer	Invasion of pleura, NOS	Pulmonary ligament	Extension to:Pleura, visceral or NOS (WITHOUT pleural effusion) Pulmonary ligament	Stated as T2a with no other information on extension	Stated as T2b with no other information on extension	Stated as T2 [NOS] with no other information on size or extension	Tumor of/involving main stem bronchus less than 2.0 cm from carina	Tumor of/involving main stem bronchus less than 2.0 cm from carina + Atelectasis/obstructive pneumonitis that extends to the hilar region but does not involve the entire lung Or atelectasis/obstructive pneumonitis, NOS	Extension to:Pleura, visceral or NOS (WITHOUT pleural effusion) Pulmonary ligament + Tumor of/involving main stem bronchus less than 2.0 cm from carina	Tumor of/involving main stem bronchus less than 2.0 cm from carina + Invasion of pleura	Atelectasis/obstructive pneumonitis involving entire lung	Parietal pericardium or pericardium, NOS	Stated as T3 with no other information on extension	Invasion of phrenic nerve	Direct extension to:Brachial plexus, inferior branches or NOS, from superior sulcus/Chest (thoracic) wall/Diaphragm/Pancoast tumor (superior sulcus syndrome), NOS/Parietal pleura	Superior sulcus tumor WITH encasement of subclavian vessels OR WITH unequivocal involvement of superior branches of brachial plexus	Multiple masses/separate tumor nodule(s) in the SAME lobe	Tumor confined to carina	Blood vessel(s), major	Blood vessel(s), major + Direct extension to:Brachial plexus, inferior branches or NOS, from superior sulcus/Chest (thoracic) wall/Diaphragm/Pancoast tumor (superior sulcus syndrome), NOS/Parietal pleura/Superior	Heart/Visceral pericardium	Malignant pleural effusion	Adjacent rib	Aorta	Aorta + Heart/Visceral pericardium	Aorta + Adjacent rib	Vertebra€/Neural foramina	Pleural tumor foci separate from direct pleural invasion	Inferior vena cava	Adjacent rib +other extention	Adjacent rib + Blood vessel(s), major	Malignant pericardial effusion	Stated as T4 with no other information on extension	Further contiguous extension	Stated as T4 with no other information on extension	Tumor proven by presence of malignant cells in sputum or bronchial washings but not visualized by imaging or bronchoscopy; "occult" carcinoma
CS mets at dx (2004+)	Stated as M1a with no other information on distant metastasis	Distant lymph node(s), including cervical nodes	Distant lymph nodes plus pleural or pericardial effusion	Distant lymph nodes plus pleural tumor foci	Separate tumor nodule(s) in different lobe, same lung	Distant lymph nodes plus extension to contralateral lung	Extension to:Skeletal muscle/Sternum/Skin of chest	Extension to:Skeletal muscle/Sternum/Skin of chest +Contralateral lung/Contralateral main stem bronchus/Separate tumor nodule(s) in contralateral lung/Pleural tumor foci or nodules on contralateral lung	Extension to:Contralateral lung/Contralateral main stem bronchus/Separate tumor nodule(s) in contralateral lung	Abdominal organs	Abdominal organs+Contralateral lung/Contralateral main stem bronchus/Separate tumor nodule(s) in contralateral lung/Pleural tumor foci or nodules on contralateral lung	Distant metastasis plus pleural or pericardial effusion	Distant metastasis plus pleural tumor foci	Distant metastases + Distant node(s) + Contralateral lung/Contralateral main stem bronchus/Separate tumor nodule(s) in contralateral lung	Distant metastasis plus distant lymph node(s)	Distant metastasis plus distant lymph nodes plus pleural or pericardial effusion	Distant metastases plus distant lymph nodes plus pleural tumor foci	Stated as M1b with no other information on distant metastasis	Stated as M1 [NOS] with no other information on distant metastasis																						

© 2020 She et al. *JAMA Network Open.*

eTable 2. Characteristics of Patients in the Training Dataset of Survival Analysis

Stage	IA1		IA2		IA3		IB		IIA		IIB		IIIA		IIIB		IIIC		IVA		IVB		total	
	median	range	median	range	median	range	median	range	media	range	medi	range	median	range	media	range	media	range	media	range	median	range	median	range
Age at diagnosis	67	36-90	67	29-90	69	31-92	69	32-92	69	35-86	67	30-95	67	29-90	66	34-89	64	46-85	65	28-84	61	50-78	68	28-95
	count	%	count	%	count	%	count	%	count	%	count	%	count	%	count	%	count	%	count	%	count	%	count	%
Sex																								
Female	324	62.5	1585	57.5	1079	56.7	1158	51.3	180	45	1025	44.9	908	47	213	42.9	5	29.4	175	50.7	5	62.5	6657	51.6
Male	194	37.5	1172	42.5	823	43.3	1098	48.7	220	55	1258	55.1	1022	53	283	57.1	12	70.6	170	49.3	3	37.5	6255	48.4
Histologic Type 2																								
AD	410	79.2	2110	76.5	1347	70.8	1492	66.1	223	55.8	1384	60.6	1245	64.5	307	61.9	10	58.8	260	75.4	6	75	8794	68.1
SD	108	20.8	647	23.5	555	29.2	764	33.9	177	44.3	899	39.4	685	35.5	189	38.1	7	41.2	85	24.6	2	25	4118	31.9
Marital status at diagnosis																								
un-married	203	39.2	1149	41.7	805	42.3	970	43	163	40.8	931	40.8	753	39	191	38.5	4	23.5	133	38.6	2	25	5304	41.1
married	315	60.8	1608	58.3	1097	57.7	1286	57	237	59.3	1352	59.2	1177	61	305	61.5	13	76.5	212	61.4	6	75	7608	58.9
T																								
T1a	518	100	0	0	0	0	0	0	0	0	22	1	21	1.1	0	0	0	0	2	0.6	0	0	563	4.4
T1b	0	0	2757	100	0	0	0	0	0	0	219	9.6	146	7.6	5	1	0	0	29	8.4	0	0	3156	24.4
T1c	0	0	0	0	1902	100	0	0	0	0	217	9.5	182	9.4	4	0.8	0	0	36	10.4	1	12.5	2342	18.1
T2a	0	0	0	0	0	0	2256	100	0	0	499	21.9	425	22	4	0.8	0	0	73	21.2	1	12.5	3258	25.2
T2b	0	0	0	0	0	0	0	0	400	100	111	4.9	71	3.7	1	0.2	0	0	11	3.2	0	0	594	4.6
T3	0	0	0	0	0	0	0	0	0	0	1214	53.2	381	19.7	284	57.3	11	64.7	102	29.6	2	25	1994	15.4
T4	0	0	0	0	0	0	0	0	0	0	1	0	704	36.5	198	39.9	6	35.3	92	26.7	4	50	1005	7.8
N																								
N0	518	100	2757	100	1902	100	2256	100	400	100	1216	53.3	484	25.1	0	0	0	0	177	51.3	2	25	9712	75.2
N1	0	0	0	0	0	0	0	0	0	0	1067	46.7	601	31.1	0	0	0	0	63	18.3	1	12.5	1732	13.4
N2	0	0	0	0	0	0	0	0	0	0	0	0	845	43.8	482	97.2	0	0	92	26.7	3	37.5	1422	11
N3	0	0	0	0	0	0	0	0	0	0	0	0	0	0	14	2.8	17	100	13	3.8	2	25	46	0.4
M																								
M0	518	100	2757	100	1902	100	2256	100	400	100	2283	100	1930	100	496	100	17	100	0	0	0	0	12559	97.3
M1a	0	0	0	0	0	0	0	0	0	0	0	0	0	0	0	0	0	0	143	41.4	0	0	143	1.1
M1b	0	0	0	0	0	0	0	0	0	0	0	0	0	0	0	0	0	0	202	58.6	0	0	202	1.6
M1c	0	0	0	0	0	0	0	0	0	0	0	0	0	0	0	0	0	0	0	0	8	100	8	0.1
LCCS																								
Alive	496	95.8	2577	93.5	1701	89.4	1950	86.4	340	85	1758	77	1303	67.5	279	56.3	7	41.2	169	49	1	12.5	10581	81.9
dead	22	4.2	180	6.5	201	10.6	306	13.6	60	15	525	23	627	32.5	217	43.8	10	58.8	176	51	7	87.5	2331	18.1
Pleural/Elastic Layer Invasion																								
PL0	518	100	2757	100	1902	100	1369	60.7	399	99.8	1715	75.1	1450	75.1	327	65.9	14	82.4	239	69.3	8	100	10698	82.9
PL1	0	0	0	0	0	0	489	21.7	1	0.3	221	9.7	181	9.4	67	13.5	1	5.9	37	10.7	0	0	997	7.7
PL2	0	0	0	0	0	0	398	17.6	0	0	157	6.9	194	10.1	57	11.5	1	5.9	43	12.5	0	0	850	6.6
PL3	0	0	0	0	0	0	0	0	0	0	190	8.3	105	5.4	45	9.1	1	5.9	26	7.5	0	0	367	2.8
Separate Tumor Nodules																								
1	518	100	2757	100	1902	100	2255	100	400	100	1903	83.4	1617	83.8	348	70.2	14	82.4	241	69.9	3	37.5	11958	92.6
2	0	0	0	0	0	0	0	0	0	0	0	0	158	8.2	47	9.5	0	0	42	12.2	3	37.5	250	1.9
3	0	0	0	0	0	0	1	0	0	0	380	16.6	126	6.5	91	18.3	2	11.8	45	13	1	12.5	646	5
4	0	0	0	0	0	0	0	0	0	0	0	0	29	1.5	10	2	1	5.9	17	4.9	1	12.5	58	0.4
Surgery to Other Regional/Distant Sites																								
None	515	99.4	2748	99.7	1892	99.5	2236	99.1	399	99.8	2252	98.6	1885	97.7	480	96.8	14	94.1	275	79.7	7	87.5	12705	98.4
Distant	0	0	4	0.1	4	0.2	7	0.3	0	0	11	0.5	11	0.6	4	0.8	0	0	64	18.6	1	12.5	106	0.8
Regional	3	0.6	5	0.2	6	0.3	13	0.6	1	0.3	20	0.9	34	1.8	12	2.4	1	5.9	6	1.7	0	0	101	0.8

© 2020 She et al. *JAMA Network Open.*

eTable 3. Characteristics of Patients in the Test Dataset of Survival Analysis

Stage	IA1		IA2		IA3		IB		IIA		IIB		IIIA		IIIB		IIIC		IVA		IVB		total	
	median	range	median	range	median	range	median	range	median	range	median	range	median	range	median	range	median	range	median	range	median	range	median	range
Age at diagnosi	68	34-86	67	35-90	69	36-92	69	29-90	68	40-87	68	42-91	67	39-88	66	19-87	63	36-68	65	40-83	67	59-69	68	19-92
	count	%	count	%	count	%	count	%	count	%	count	%	count	%	count	%	count	%	count	%	count	%	count	%
Sex																					3		3228	
Female	82	66.1	407	57.2	267	52	278	50.9	35	35.4	267	50.1	203	41.7	51	44	2	66.7	45	48.4	2	66.7	1639	50.8
Male	42	33.9	304	42.8	246	48	268	49.1	64	64.6	266	49.9	284	58.3	65	56	1	33	48	51.6	1	33.3	1589	49.2
Histologic Type 2																								
AD	99	79.8	541	76.1	369	71.9	380	69.6	48	48.5	346	64.9	319	65.5	73	62.9	2	66.7	64	68.8	2	66.7	2243	69.5
SD	25	20.2	107	23.9	144	28.1	166	30.4	51	51.5	187	35.1	168	34.5	43	37.1	1	33.3	29	31.2	1	33.3	985	30.5
Marital status at diagnosis																								
married	59	47.6	308	43.3	294	57.3	305	55.9	40	40.4	295	55.3	295	60.6	69	59.5	1	33.3	56	60.2	1	33.3	1843	57.1
un-married	65	52.4	403	56.7	219	42.7	241	44.1	59	59.6	238	44.7	192	39.4	47	40.5	2	66.7	37	39.8	2	66.7	1385	42.9
T																								
T1a	124	100	0	0	0	0	0	0	0	0	7	1.3	7	1.4	0	0	0	0	4	1.1	0	0	139	4.3
T1b	0	0	711	100	0	0	0	0	0	0	39	7.3	43	8.8	2	1.7	0	0	9	9.7	0	0	804	24.9
T1c	0	0	0	0	513	100	0	0	0	0	65	12.2	52	10.7	2	1.7	0	0	9	9.7	0	0	641	19.9
T2a	0	0	0	0	0	0	546	100	0	0	126	23.6	97	19.9	2	1.7	0	0	20	21.5	0	0	791	24.5
T2b	0	0	0	0	0	0	0	0	99	100	18	3.4	19	3.9	0	0	0	0	5	5.4	0	0	141	4.4
T3	0	0	0	0	0	0	0	0	0	0	278	52.2	76	15.6	67	57.8	1	33.3	22	23.7	1	33.3	445	13.8
T4	0	0	0	0	0	0	0	0	0	0	0	0	193	39.6	43	37.1	2	66.7	27	29	2	66.7	267	8.3
N																								
N0	124	100	711	100	513	100	546	100	99	100	278	52.2	120	24.6	0	0	0	0	47	50.5	1	33.3	2439	75.6
N1	0	0	0	0	0	0	0	0	0	0	255	47.8	149	30.6	0	0	0	0	13	14	1	33.3	418	12.9
N2	0	0	0	0	0	0	0	0	0	0	0	0	218	44.8	110	94.8	0	0	27	29	1	33.3	356	11
N3	0	0	0	0	0	0	0	0	0	0	0	0	0	0	6	5.2	4	100	6	6.5	0	0	15	0.5
M																								
M0	124	100	711	100	513	100	546	100	99	100	533	100	487	100	116	100	3	100	0	0	0	0	3132	97
M1a	0	0	0	0	0	0	0	0	0	0	0	0	0	0	0	0	0	0	41	44.1	0	0	41	1.3
M1b	0	0	0	0	0	0	0	0	0	0	0	0	0	0	0	0	0	0	52	55.9	0	0	52	1.6
M1c	0	0	0	0	0	0	0	0	0	0	0	0	0	0	0	0	0	0	0	0	3	100	3	0.1
LCCS																								
Alive	121	97.6	657	92.4	470	91.6	478	87.5	91	91.9	413	77.5	321	65.9	66	56.9	1	33.3	47	50.5	1	33.3	2666	82.6
dead	3	2.4	54	7.6	43	8.4	68	12.5	8	8.1	120	22.5	166	34.1	50	43.1	2	66.7	46	49.5	2	66.7	562	17.4
Pleural/Elastic Layer Invasion																								
PL0	124	100	711	100	513	100	315	57.7	99	100	405	76	360	73.9	80	69	2	66.7	66	71	1	33.3	2676	82.9
PL1	0	0	0	0	0	0	133	24.4	0	0	47	8.8	50	10.3	10	8.6	0	0	8	8.6	1	33.3	249	7.7
PL2	0	0	0	0	0	0	98	17.9	0	0	37	6.9	52	10.7	17	14.7	1	33.3	15	16.1	1	33.3	221	6.8
PL3	0	0	0	0	0	0	0	0	0	0	44	8.3	25	5.1	9	7.8	0	0	4	4.3	0	0	82	2.5
Separate Tumor Nodules																								
1	124	100	711	100	513	100	546	100	99	100	433	83.1	410	84.2	82	70.7	3	100	69	74.2	2	66.7	3002	93
2	0	0	0	0	0	0	0	0	0	0	0	0	36	7.4	10	8.6	0	0	10	10.8	1	33.3	57	1.8
3	0	0	0	0	0	0	0	0	0	0	90	16.9	28	5.7	23	19.8	0	0	10	10.8	0	0	151	4.7
4	0	0	0	0	0	0	0	0	0	0	0	0	13	2.7	1	0.9	0	0	4	4.3	0	0	18	0.6
Surgery to Other Regional/Distant Sites																								
None	124	100	711	100	513	100	545	99.8	99	100	527	98.9	478	98.2	115	99.1	3	100	74	79.6	2	66.7	3181	98.5
Distant	0	0	0	0	0	0	0	0	0	0	0	0	2	0.4	0	0	0	0	17	18.3	1	33.3	24	0.7
Regional	0	0	0	0	0	0	1	0.2	0	0	6	1.1	7	1.4	1	0.9	0	0	2	2.2	0	0	23	0.7

© 2020 She et al. *JAMA Network Open.*

eTable 4. Feature Component Weightings in the DeepSurv Model

Features	Weight	Features	Weight	Features	Weight
Age at diagnosis	0.5722479	CS extension (2004+)=540	0.42365953	Stage=3	-0.0717
CS tumor size (2004+)	0.6724694	CS extension (2004+)=550	0.08433475	Stage=4	-0.07168
Regional nodes examined (1988+)	-0.4995487	CS extension (2004+)=560	0.13039102	Stage=5	-0.01987
Regional nodes positive (1988+)	0.7574372	CS extension (2004+)=570	0.06113536	Stage=6	0.041379
Sex=1	-0.062900014	CS extension (2004+)=590	0.22531554	Stage=7	0.101898
Sex=2	0.1671767	CS extension (2004+)=600	0.12043501	Stage=8	0.093093
Histologic Type ICD-O-3=0	0.055728845	CS extension (2004+)=610	-0.90538454	Stage=9	0.104782
Histologic Type ICD-O-3=1	-0.048216447	CS extension (2004+)=680	-0.824522	Stage=10	0.034815
Histologic Type ICD-O-3=2	0.015860233	CS extension (2004+)=700	0.16996412	Stage=11	0.51159
Histologic Type ICD-O-3=3	-0.08446639	CS extension (2004+)=705	-0.9847599	T8=1	0.209211
Histologic Type ICD-O-3=4	0.0417398	CS extension (2004+)=710	0.3103478	T8=2	0.038959
Histologic Type ICD-O-3=5	-0.00887268	CS extension (2004+)=730	-0.20042646	T8=3	0.056467
Histologic Type ICD-O-3=6	0.025923487	CS extension (2004+)=740	0.81883913	T8=4	0.049242
Histologic Type 2=1	-0.028282069	CS extension (2004+)=745	0.40200815	T8=5	0.06238
Histologic Type 2=2	0.020090567	CS extension (2004+)=750	0.16024342	T8=6	-0.09599
Grade=1	-0.002670259	CS extension (2004+)=770	-0.86313397	T8=7	-0.12861
Grade=2	0.063437365	CS extension (2004+)=785	-0.17053518	N8=1	-0.06939
Grade=3	0.15810749	CS mets at dx (2004+)=0	-0.07108595	N8=2	0.052847
Grade=4	-0.124901354	CS mets at dx (2004+)=15	0.14313275	N8=3	0.070122
RX Summ--Scope Reg LN Sur (2003	-0.05786139	CS mets at dx (2004+)=16	0.08157881	N8=4	0.202186
RX Summ--Scope Reg LN Sur (2003	0.029179208	CS mets at dx (2004+)=17	-0.34559816	M8=1	-0.21266
RX Summ--Scope Reg LN Sur (2003	0.020770853	CS mets at dx (2004+)=18	0.059344094	M8=2	-0.02519
RX Summ--Scope Reg LN Sur (2003	-0.03520994	CS mets at dx (2004+)=20	-0.12556794	M8=3	0.030704
CS extension (2004+)=100	-0.0725175	CS mets at dx (2004+)=21	0.99101025	M8=4	0.576268
CS extension (2004+)=110	0.04731824	CS mets at dx (2004+)=23	-0.19575515	Marital status at diagnosis=0	0.034368
CS extension (2004+)=115	0.026038347	CS mets at dx (2004+)=24	-0.037997384	Marital status at diagnosis=1	-0.10559
CS extension (2004+)=120	0.004864246	CS mets at dx (2004+)=25	-0.76219594	Lung - Pleural/Elastic Layer Invasion (PL) by H and E or Elastic Stain=0	0.001433
CS extension (2004+)=125	-0.09474413	CS mets at dx (2004+)=26	0.90065193	Lung - Pleural/Elastic Layer Invasion (PL) by H and E or Elastic Stain=1	0.034629
CS extension (2004+)=200	0.021685144	CS mets at dx (2004+)=30	-0.18796362	Lung - Pleural/Elastic Layer Invasion (PL) by H and E or Elastic Stain=2	0.001899
CS extension (2004+)=210	-0.16040157	CS mets at dx (2004+)=32	0.6542427	Lung - Pleural/Elastic Layer Invasion (PL) by H and E or Elastic Stain=3	0.164463
CS extension (2004+)=220	-0.10082796	CS mets at dx (2004+)=33	-1.1801782	Lung - Separate Tumor Nodules - Ipsilateral Lung=1	-0.03875
CS extension (2004+)=230	-0.21979994	CS mets at dx (2004+)=36	0.88033414	Lung - Separate Tumor Nodules - Ipsilateral Lung=2	0.063924
CS extension (2004+)=300	-0.033153117	CS mets at dx (2004+)=37	-0.1698591	Lung - Separate Tumor Nodules - Ipsilateral Lung=3	0.042076
CS extension (2004+)=400	-0.029358057	CS mets at dx (2004+)=40	0.14898834	Lung - Separate Tumor Nodules - Ipsilateral Lung=4	0.150139
CS extension (2004+)=410	-0.102100626	CS mets at dx (2004+)=41	0.5691616	Lung - Surgery to Primary Site (1988-2015)=1	0.005233
CS extension (2004+)=420	-0.07669448	CS mets at dx (2004+)=42	0.032155376	Lung - Surgery to Primary Site (1988-2015)=2	-0.13327
CS extension (2004+)=430	0.057814617	CS mets at dx (2004+)=43	0.030705813	Lung - Surgery to Primary Site (1988-2015)=3	-0.03041
CS extension (2004+)=440	0.22026922	CS mets at dx (2004+)=51	0.6830787	Lung - Surgery to Primary Site (1988-2015)=4	0.141988
CS extension (2004+)=455	-0.01990803	CS mets at dx (2004+)=52	1	Lung - Surgery to Other Regional/Distant Sites (1998+)=1	-0.04313
CS extension (2004+)=460	-0.061044298	CS mets at dx (2004+)=53	0.6274554	Lung - Surgery to Other Regional/Distant Sites (1998+)=2	0.117256
CS extension (2004+)=465	0.56912345	CS mets at dx (2004+)=70	-0.58639836	Lung - Surgery to Other Regional/Distant Sites (1998+)=3	0.06074
CS extension (2004+)=500	-0.10761972	Stage=1	-0.4348911		
CS extension (2004+)=520	0.20671241	Stage=2	-0.15681928		

© 2020 She et al. *JAMA Network Open.*

eTable 5. Survival Predictors in Cox PH Model

Variables	HR	95% CI	*β*	*P* value
Age at diagnosis	1.022	1.017-1.026	0.022	<0.001
Sex	1.429	1.310-1.558	0.357	<0.001
Histologic	1.151	1.053-1.258	0.141	0.002
Grade	0.911	0.869-0.955	-0.093	<0.001
RX Summ-Scope Reg LN Sur (2003+)	0.895	0.825-0.972	-0.111	0.008
CS tumor size (2004+)	1.009	1.006-1.011	0.009	<0.001
CS extension (2004+)	1.039	1.008-1.071	0.039	0.013
CS mets at dx (2004+)	0.973	0.948-0.999	-0.027	0.039
Regional nodes examined (1988+)	0.987	0.981-0.992	-0.013	<0.001
Regional nodes positive (1988+)	1.053	1.035-1.071	0.051	<0.001
Stage	1.272	1.214-1.334	0.241	0.001
T stage	0.926	0.881-0.974	-0.076	0.003
N stage	1.148	1.055-1.248	0.138	0.001
M stage	2.066	1.240-3.443	0.726	0.005
Marital status	0.791	0.727-0.861	-0.234	<0.001
Lung-Pleural/Elastic Layer Invasion (PL) by H and E or Elastic Stain	1.109	1.044-1.179	0.104	0.001

Cox PH: Cox proportional hazard regression; HR: Hazard Ratio; CI: Confidence Interval. Hx=0.022 * Age at diagnosis+ 0.357 * Sex + 0.141 * Histologic- Grade * 0.093 - 0.111 * RX Summ-Scope Reg LN Sur (2003+) + 0.009 * CS tumor size (2004+) + 0.039 * CS extension (2004+) - 0.027 * CS mets at dx (2004+) - 0.013 * Regional nodes examined (1988+) + 0.051 * Regional nodes positive (1988+) + 0.241 * Stage - 0.076 * T stage+ 0.138 * N stage + 0.726 * M stage - 0.234 * Marital status + 0.104 * Lung - Pleural/Elastic Layer Invasion (PL) by H and E or Elastic Stain.

© 2020 She et al. *JAMA Network Open.*

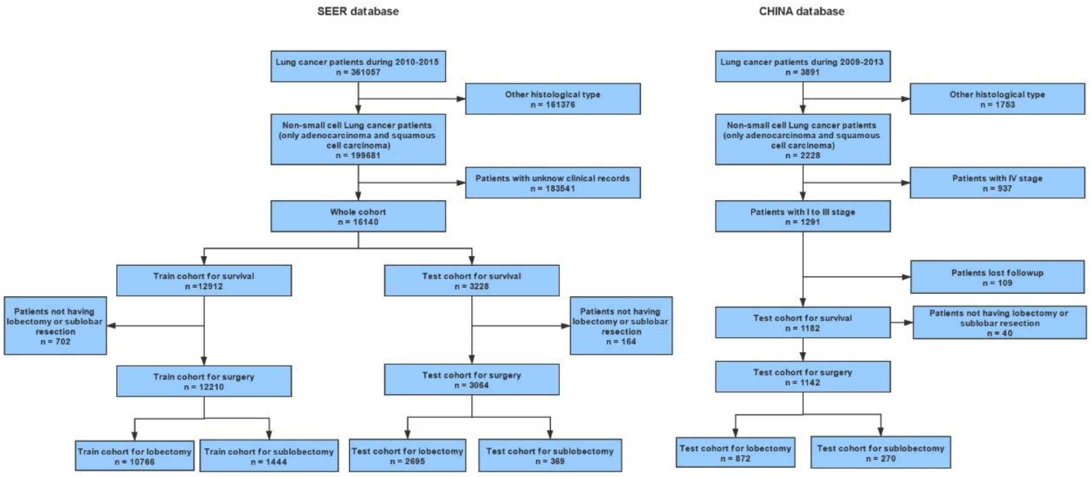

eFigure 1. Flow chart of datasets construction. (A) SEER dataset, (B) CHINA dataset

© 2020 She et al. *JAMA Network Open.*

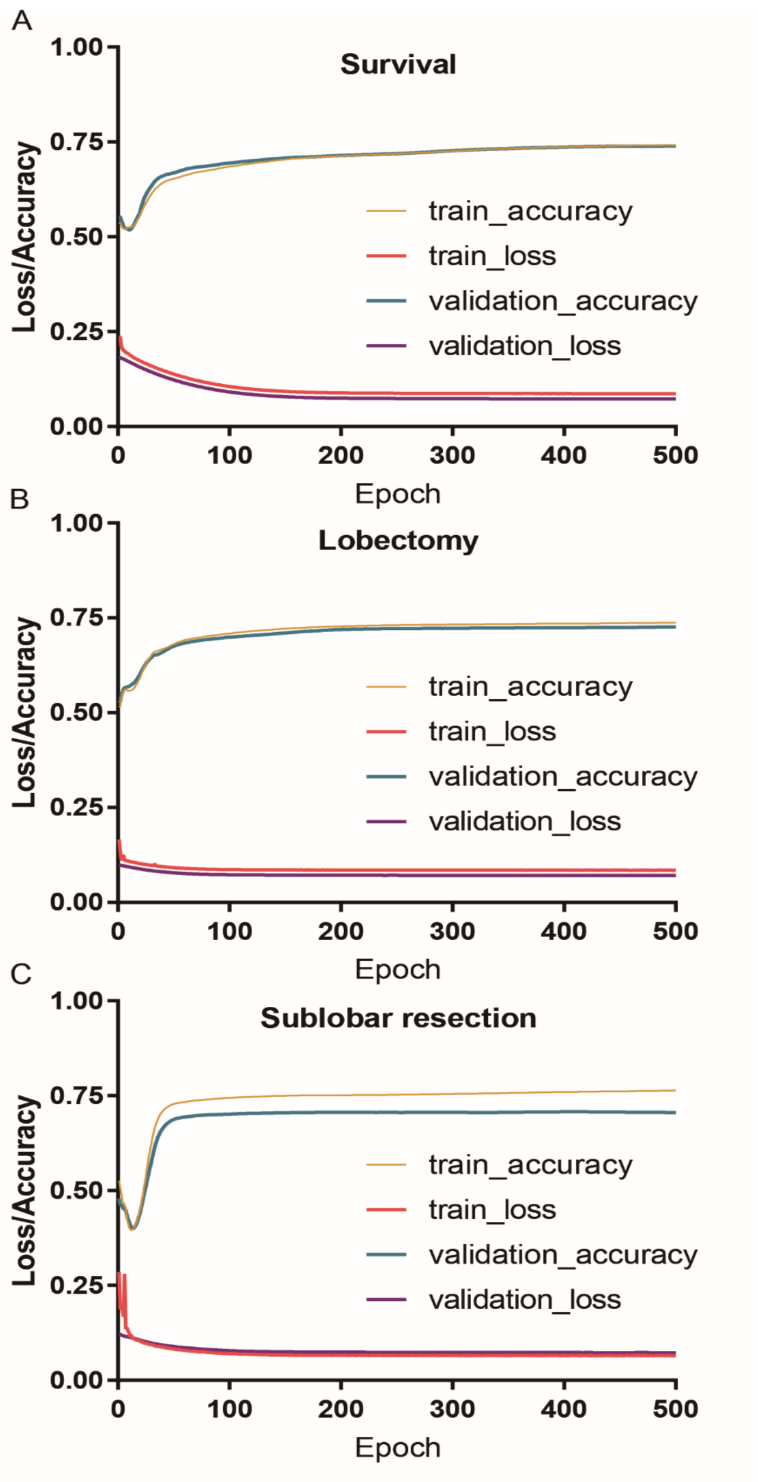

eFigure 2. Training curves of networks in the survival dataset of SEER database (A), lobectomy dataset (B), and sublobar resection dataset (C). The red and purple curves indicate loss of the training and test datasets, respectively; the blue and yellow curves indicate the accuracy of the training and test datasets, respectively.

© 2020 She et al. *JAMA Network Open.*

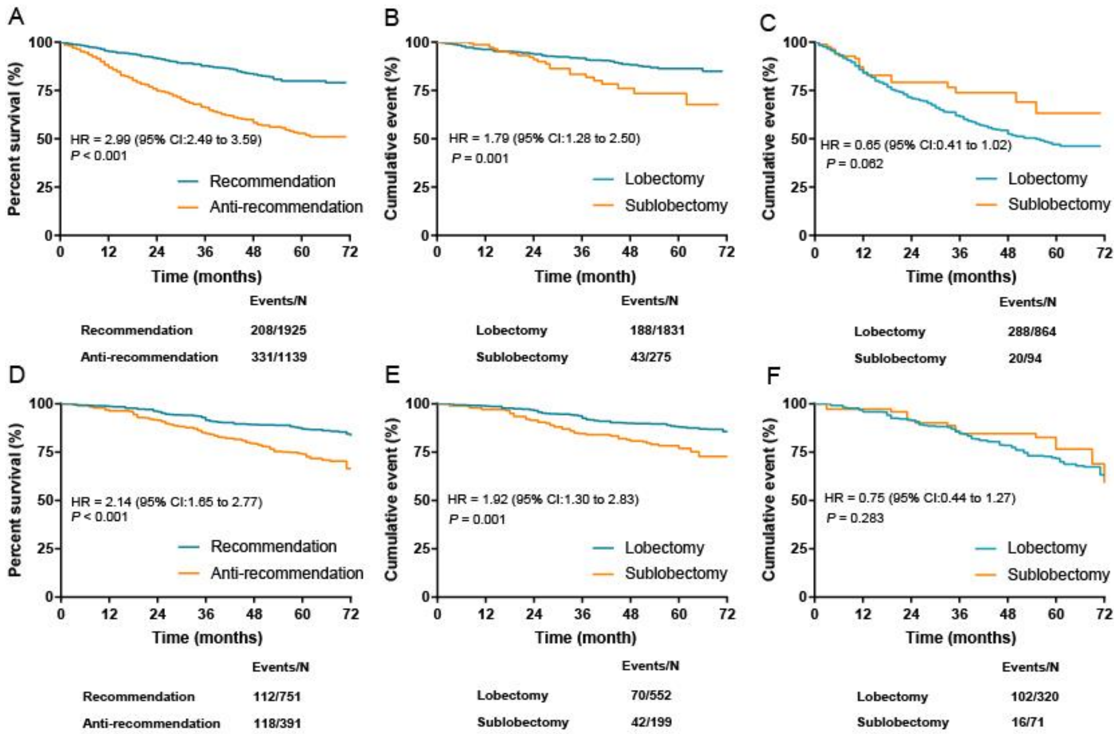

eFigure 3. Lung cancer–specific survival recommendation comparisons of SEER dataset (A), SEER lobectomy test dataset (B), and SEER sublobar resection test dataset (C); Lung cancer–specific survival recommendation comparisons of CHINA dataset (D), CHINA lobectomy test dataset (E), and CHINA sublobar resection test dataset (F).

© 2020 She et al. *JAMA Network Open.*
